# 1008. Case Study. Chips and Dips: Novel Application of Silver Dressing for Chronic Burn Wounds

**DOI:** 10.1093/jbcr/irag033.196

**Published:** 2026-04-06

**Authors:** Philip H Chang, Lara Bennett

**Affiliations:** William Randolph Hearst Burn Center, NY, New York; William Randolph Hearst Burn Center, West Point, NY

## Abstract

**Patient Presentation (age range, injury details, relevant history):**

18 year old woman suffered 60% total body surface area mixed depth partial thickness burns from a gas stove explosion in a recreational vehicle. She had undergone multiple surgical procedures to excise and graft her burn wounds and had achieved 90% closure within 45 days. However, the remaining wounds did not close with conventional daily topical therapy and were deemed too small in size to surgically graft. The patient had no significant past medical history or past surgical history.

**Clinical Challenges:**

Her scattered wounds varied in size from less than 1 cm in diameter to a maximum of 3 cm in diameter and were scattered over bilateral lower extremities and her back. The persisting chronic open wounds were causing the patient tremendous discomfort, and as her hospital course progressed, she occasionally refused daily wound care.

**Management Approach:**

From previous experience with chronic open wounds at the post-grafting stage, our team applied "chips" made of a single layer of a well-known nanocrystalline silver dressing. The chips are applied and cut to size to cover the scattered open wounds (nicknamed "dips") and then dressed with dry dressings over the "chips" covering the "dips." The "chips" eventually dry and adhere to the granulation tissue of the open wounds, providing a temporary outer coverage that remains in place until re-epithelialization occurs underneath the "chips." This eliminates the need for daily wound care and significantly decreases the discomfort of the patient.

**Outcomes:**

After 3 weeks of "chips" therapy to cover the "dips," the patient had achieved over 99% closure and was deemed ready to discharge to acute inpatient rehabilitation. In subsequent outpatient follow-up, the previously open wounds remained closed.

**Lessons Learned:**

"Chips" of the nanocrystalline silver dressing can provide an alternative dressing for small chronic open burn wounds especially after multiple rounds of skin grafting.

**Applicability to Practice:**

This has applicability for clinical practice especially for patients who suffer from persisting open wounds despite an extended period of conventional wound care after skin grafting procedures.

